# A systematic review of the measures that have been used to assess surface characteristics in relation to their impact on walking and falling

**DOI:** 10.1186/s13643-023-02315-0

**Published:** 2023-09-02

**Authors:** Rebecca Lawson, Nicholas D. A. Thomas

**Affiliations:** 1 Institute of Population Health, University of Liverpool, Liverpool, L69 7ZA UK; 2 Institute of Life Course & Medical Sciences, University of Liverpool, Liverpool, L7 8TX UK

**Keywords:** Locomotion, Gait, Surfaces

## Abstract

We investigated whether an impediment to progress in understanding the environmental factors that cause falls may be the difficulty in comparing results across studies because walking surfaces are poorly defined and underspecified. We conducted a systematic review of 384 studies from 370 articles that tested how different surfaces influenced human walking and falling. For each study, we report which categories of surfaces were used (indoor, outdoor, treadmill, virtual reality and qualitative), the nature of each surface (stairs, slopes, slippery, compliant, rough or default) and how information about each surface was measured. We found that minimal information was provided for many surfaces, making it impossible to meaningfully compare results for different types of surfaces across studies. We conclude that most published studies of walking and falling provide insufficient data to describe the surfaces that they used and we provide recommendations about how to improve the reporting of walking surfaces.

**Supplementary Information:**

The online version contains supplementary material available at 10.1186/s13643-023-02315-0.

## Introduction

Indoor and outdoor falls often result in major physical and emotional burdens on the individuals involved and their families, and they place a significant financial burden on health providers [13]. Two types of factors cause falls: individual and environmental. Accordingly, fall risk can be reduced by individual interventions (e.g. strength and balance training) or by environmental interventions (e.g. replacing worn carpet). A large body of research has investigated individual fall risk factors (see reviews by Berg and Cassells [2], Hopewell et al. [10]. Less research has addressed environmental fall risk factors and interventions. Identifying techniques to reduce environmental fall risks would complement individual fall prevention training programmes.

Research on environmental fall risk factors may be being held back because researchers have not agreed on what aspects of the environment need to be measured and how to do this. This paper aims to address this issue by focussing on an important subset of environmental fall risk factors that can be assessed using surface measurements. The nature of the walking surface (e.g. if it is slippery, rough or steeply sloping) influences walking and the risk of falls. However, there is not, as yet, a consensus in the literature as to how to measure such surface qualities [17]. Furthermore, studies investigating the environmental factors that affect walking and falling often report minimal information about the surfaces used, making it difficult to compare results across studies. For example, Matthis, Yates, & Hayhoe [11] reported a technically impressive study that precisely measured both gaze location and body position as people walked outdoors on flat, medium and rough surfaces. However, the surfaces themselves were only described verbally. In this paper, we present a systematic review that assessed how surface qualities have been reported in the extant literature on walking and fall risk in order to authoritatively evaluate current practise. Our goal was to highlight problems and to provide advice about improving the reporting of surface factors in research on fall risk.

Many of the factors influencing the walkability of surfaces are poorly defined within the literature on walking and falling. Within this paper, we used the following definitions, ordered from best to least well defined:

- *Stairs* refer to a series of perpendicular surfaces that alternate between vertical (the rise, typically ~ 0.2m) and horizontal (the going, typically ~ 0.25m) and that can be either ascended or descended.
- *Slope* is a (positive or negative) change in height between the start and end of a walking surface (for up slopes and down slopes, respectively). Surfaces may also slope in other directions, for example perpendicular to the direction of walking (cross slopes). Zero slope surfaces are horizontal. Slope (i.e. large-scale changes in surface height) is independent of surface roughness (i.e. small-scale changes in surface height), so a given slope can be either rough or smooth.
- *Slipperiness* is the coefficient of kinetic friction. This can be calculated as a surface property of an untreated surface (such as dry concrete). It can also be calculated for a surface after adding a contaminant (such as water, oil or glycerol) in which case slipperiness arises from a combination of the surface plus contaminant.
- *Compliance* refers to the elastic deformation (or springiness or flexibility) that occurs when a force is applied to a surface. Compliance is low for concrete slabs and high for foam mats.
- *Roughness* (or unevenness or texture; the opposite of roughness is smoothness) refers to small-scale variation in the height of a surface and it is independent of slope. Smooth surfaces have no depth variation whereas rough surfaces have variation in the scale range of millimetres (for depth changes that can be detected under a single foot) up to approximately one metre (for depth changes that can be detected from one stride to the next).
- *Default* surface is smooth with zero slope, and it does not belong to any of the above five types, so it is not slippery or springy or stairs. We suggest that the term flat should be avoided to describe surfaces because it is ambiguous: it could refer to a zero-slope surface and/or to a smooth surface. We included this special, combination category of surface because it was so frequently used in studies. Default surfaces are also commonplace in everyday life. For example, the overwhelming majority of surfaces throughout public buildings (and in laboratories) are default.

## Methodology for the systematic review

In this section, we describe our method for conducting a systematic review of the published studies investigating walking and falling in relation to different surfaces. This review was conducted in order to determine what measures have been used to assess physical aspects of surfaces.

### Procedure

This systematic review is reported according to PRISMA guidelines (see Supplementary material SM1) [12]. We were only interested in the different measurements used for surfaces, and not the study outcomes, so some PRISMA guidelines criteria were not relevant and were omitted. Prior to conducting the review, a protocol was developed and registered with PROSPERO, an international prospective register of systematic reviews (REF: CRD42021222694). The systematic searches were conducted in December 2020.

### Eligibility

#### Study type

Articles were only included if they reported trial studies, observational studies, reviews that included previously unpublished data, before and after studies, small case studies and conference papers. We excluded articles that only reported single case reports, letters and studies that did not report new data (such as meta-analyses and systematic reviews). Only articles published in the English language were considered.

### Participants and movements

We only included studies that tested at least two human participants (not animal or robot participants or purely modelling or simulation studies). There were no restrictions on participant age or health, but studies had to include an assessment of walking and/or falling. Studies were excluded if they only tested movements other than walking (e.g. running, skiing, pulling or pushing objects, avoiding obstacles, or traversing a single stair).

### Surfaces

Studies had to include at least two surfaces and the surfaces had to be compared either physically (e.g. with slope angles) or non-physically (e.g. using perceptual ratings or descriptions). Surfaces included indoor and outdoor surfaces, stairs, treadmills, surfaces simulated in virtual reality and images of walking surfaces. Studies that only tested stairs were excluded.

### Information sources and search strategy

The following electronic databases were searched: Web of Science (1900 to 2020), Scopus (1823 to 2020) and PubMed (1948 to 2020). The search terms used are listed in Table 1 with details of alternative suffixes given in Supplementary material (SM2).

**Table 1:** Search terms used for the systematic review. For inclusion a study required at least one term from each of the four lists of terms and to also not mention robots or animals

Term 1	Term 2	Term 3	Term 4
Surface	Smooth		
Floor	Uneven		
Topography	Rough	Gait	Participant
Substrate	Complex	Walk	Subject
Terrain	Irregular	Locomotion	Adults
Sidewalk	Flat	Stride	Children
Pavement	Slippery		
Ground			
OR	OR	OR	OR
AND			
AND NOT			
Animal			
Robot			

### Screening

All articles (*n* = 5218) identified from the three databases were uploaded into EndNote (X8, Clarivate Analytics, USA) bibliographic software. Duplicates articles (1733) were removed using the inbuilt duplication tool. A further 547 duplicates were identified manually leaving 2938 articles. The second author (NT) screened these articles by checking the article titles and abstracts for eligibility. This resulted in the exclusion of 2394 articles. The first author (RL) independently screened a random 10% of the 2938 articles. The screeners had 89% agreement on the decision to include or exclude an article. Cohen’s coefficient of agreement (Kappa score, [4] was 0.61 indicating substantial agreement between the two reviewers (see Supplementary material, SM3).

### Eligibility check and data extraction

**Fig. 1:**
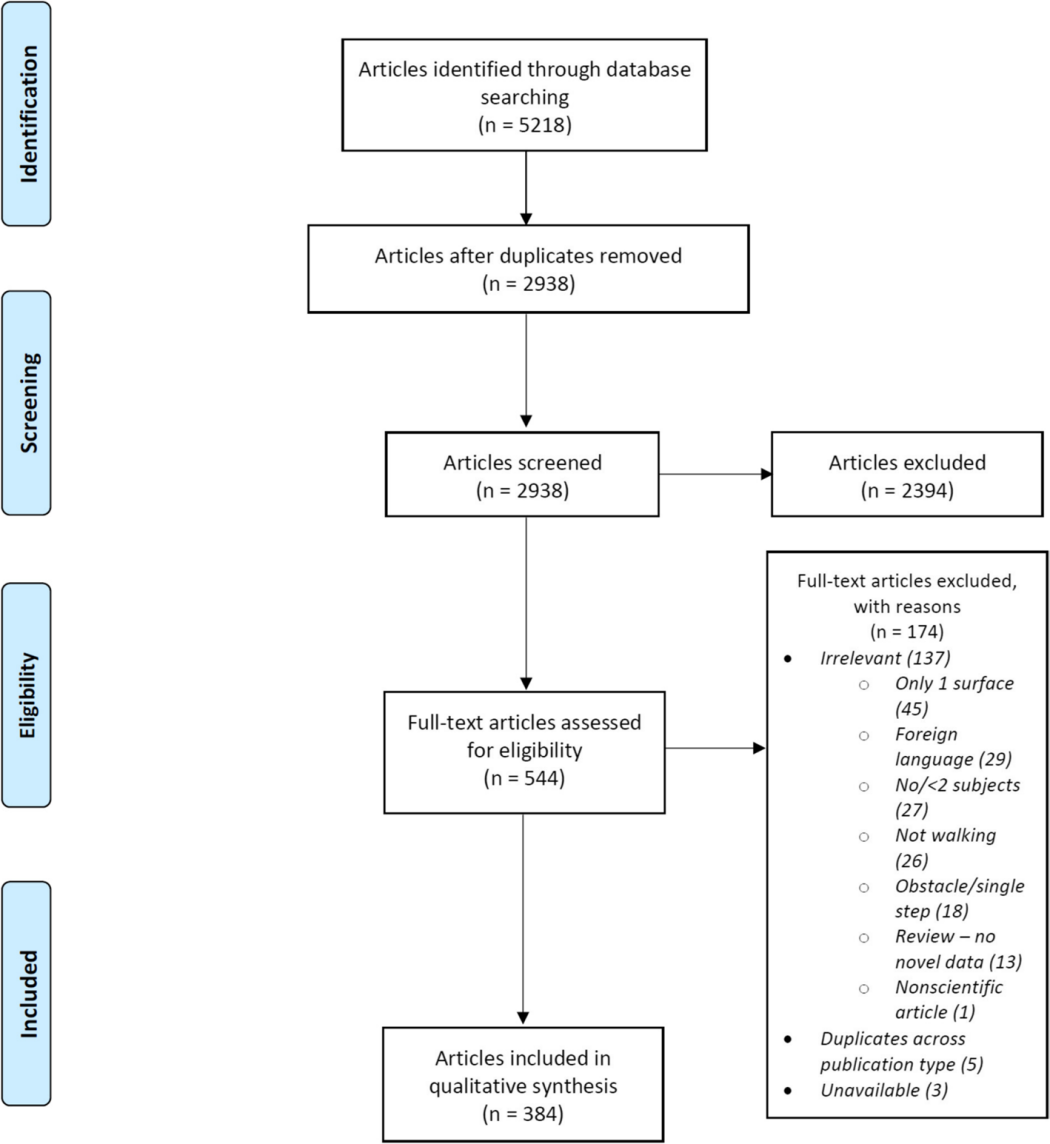
PRISMA flow diagram of articles considered for each stage of the review

NT then performed an eligibility check of the full text of the 544 articles identified as eligible after the screening. This resulted in a further 174 papers being excluded. Exclusions were mainly (*n* = 166) because the full text revealed that they did not satisfy the eligibility requirements, see Fig. 1 for details. NT then extracted into a table the data from the 384 studies reported in the remaining 370 articles (14 articles reported more than one study), see Supplementary material SM4. For each study, this table recorded the setting(s) used (indoor, outdoor, treadmill, virtual reality (VR) or qualitative such as questionnaires), the number and type of surfaces assessed (separated into stairs, slopes, slippery, compliant, rough, default and other), the physical surface measurements reported (dimensions, angles, coefficient of friction and other) and the non-physical surface measurements reported (ratings, rankings, multiple choice questions and verbal descriptions). All measures that were recorded for at least two surfaces within a study were included. We also recorded the nature and size of the population tested.

## Results

Figure 1 shows a flowchart of the progress of the systematic review. We report the results of the data extraction in the following sections: article type, participants, study settings, surface type, physical measures of surfaces and non-physical measures of surfaces.

### Study type

Of the 384 studies included, 345 were reported in journal articles (including one review), 6 were in conference reports and 33 were book chapters. The studies included experiments, focus groups and questionnaires.

### Participants

Most studies tested healthy adults (aged 18–60 or age not specified) (60%, *n* = 231), 14% (*n* = 53) tested older adults (aged over 60 or only verbally described) and just 6% (*n* = 22) tested children (aged under 18 or only verbally described). Of the remaining studies, 23% (*n* = 87) tested various clinical groups including amputees, stroke patients and people with peripheral neuropathy and Parkinson’s disease. Finally, seven studies (2%) did not identify the type of participant tested or their age. A few studies included participants from more than one of these groups.

### Settings and surfaces

There were five categories of study setting: indoor, which was used in 71% of the 384 studies, outdoor 14%, treadmill 15%, VR 4% and qualitative 12%. Note that a few studies included multiple setting types such as testing both indoors and outdoors. These categories of study setting were broken into subcategories as follows: indoor—laboratories, corridors, other indoor spaces; outdoor—pavements including tracks, fields including grass, tarmac including roads and carparks, mixed terrains; treadmill—conventional, multidirectional; VR—no subcategories; and qualitative—questionnaires, focus groups, interviews, computer-based, databases, see Table 2.

**Table 2:** Numbers of each setting type (e.g. laboratory) tested in each subcategory of setting (e.g. indoors): note that some setting types tested multiple setting subcategories (e.g. outdoors on tarmac and in a field)

Indoor	*n*	Outdoor	*n*	Treadmill	*n*	VR	*n*	Qualitative	*n*
Laboratory	245	Pavement	28	Normal	52	VR	14	Questionnaire	32
Corridor	25	Tarmac	4	Multi	6			Database	8
Other	2	Field	6					Focus group	7
Not stated	4	Mixed	18					Computer	4
		Not stated	9						
**Total**	**276**		**65**		**58**				**51**

Surfaces were categorised as stairs, slopes, slippery, compliant, rough, default and other, see Table 3. In addition, contaminants (e.g. water, oil) could be added to surfaces. Surfaces could be categorised as multiple surface types (e.g. both slippery and rough). If surface descriptions were unclear or unique, they were assigned to the “other” category. Some types of surface (e.g. rough) are less precisely defined than others (e.g. stairs). It is unclear whether the lack of a precise definition for rough surfaces means that a greater range of surfaces are included in this category or whether the intrinsic variability in rough surfaces makes it difficult to produce a satisfactory narrow definition. We return to this issue in the “Discussion” section.

**Table 3:** For each surface type (e.g. stairs), the percentage of studies that included each kind of setting (e.g. indoors, VR): surface types were often assessed in more than one setting within a given study (e.g. both indoors and outdoors) so total row values are greater than 100%

%	Indoors	Outdoors	Treadmill	VR	Questionnaire	**Total %**
Stairs	84	31	16	0	4	**135**
Slopes	68	19	26	6	1	**120**
Slippery	94	9	5	3	0	**109**
Compliant	93	0	13	0	0	**107**
Rough	75	24	14	4	2	**119**
Default	81	14	17	3	1	**116**
Other	66	47	16	3	3	**134**
**Mean %**	**80**	**21**	**15**	**3**	**2**	

### Physical surface measures

Most studies (83%; 319 of 384) included physical surface measurements. These could comprise distances (length, width, depth or combinations of these), angles (reported in degrees, percentage gradient or gradient) and coefficients of friction (COF), see Table 4. For each of the surface types, we now discuss which physical measurements were reported. Note that some surfaces were categorised in multiple ways (e.g. both length and roughness).

**Table 4:** The percentage of studies that recorded each type of physical measure for each surface type

Surface type (*n* studies)	Dimensions	Angle	COF	Other	**Any**
Stairs (55)	67	35	0	49	80
Slopes (118)	64	81	5	20	92
Slippery (102)	81	12	74	11	98
Compliant (15)	100	53	0	33	100
Rough (100)	89	16	2	17	92
Default (314)	76	29	22	14	92
Other (32)	78	6	12	16	81

#### Stairs

Of the 55 studies testing stairs, 44 (80%) included physical measures, with 37 studies (67%) reporting at least one distance and 27 studies (49%) reporting the number of stairs. Only 4 of the stair studies (7%) reported the measurement technique used. These included a slip meter, a clinometer and 3D modelling software. There were 11 studies (20%) that gave only verbal descriptions for at least one of the surfaces used.

#### Slopes

Of the 118 studies testing slopes, 109 (92%) included physical measures. There were 75 studies (64%) reporting at least one distance and 95 (81%) reporting angle. Only 21 studies (18%) reported the measurement technique used. These included measuring slope with protractors, a GPS and clinometers. The remaining 9 studies (8%) gave only verbal descriptions for at least one of the surfaces used.

### Slipperiness

One hundred of the 102 slipperiness studies (98%) included physical measures with 83 studies (81%) reporting at least one distance, 75 (74%) reporting the coefficient of friction and 12 (12%) reporting slope. Sixty-six studies (65%) reported at least one of the measurement techniques used including using force plates (*n* = 16), slipmeters (*n* = 26) and tribometers (*n* = 8). Eight studies (8%) only gave verbal descriptions for at least one of the surfaces used.

### Compliance

All 15 of the compliance studies included physical measures, with all of them reporting at least one distance, eight (53%) reporting slope, three (20%) reporting compression and one (7%) reporting hardness. Only one study reported any of the measurement techniques used, which was a durometer to assess hardness.

#### Roughness

Ninety-two of the 100 roughness studies (92%) included physical measures with 89 studies (89%) reporting at least one distance, 16 (16%) reporting slope, four reporting roughness and one reporting hardness. Only 12 studies (12%) reported any of the measurement techniques used. These included clinometers (*n* = 2), profilometers (*n* = 2) and GPS (*n* = 2). Thirty (30%) studies gave only verbal descriptions of at least one of the surfaces tested.

#### Default surfaces

Of the 314 studies that tested default surfaces, 288 (92%) included physical measures. There were 240 studies (76%) that reported at least one distance, 91 (29%) that reported slope, 68 (22%) that reported coefficient of friction, 7 (2%) reported that roughness and 3 studies (1%) that reported hardness. Only 82 studies (26%) reported the measurement techniques used with at least some of the surfaces tested. A variety of techniques were used including slip-meters, force plates and protractors. Eighty-four studies (27%) gave only verbal descriptions of at least one of the surfaces tested.

#### Other surfaces

There were 32 studies that assessed surfaces that could not be included in any of the above six categories. Some surface types were used in several of these studies including grass (*n* = 5), narrow surfaces (*n* = 4), sand (*n* = 2), snow (*n* = 2) and “random” surfaces (*n* = 2). Other surface types were unique to a single study (e.g. beam) and/or included multiple surfaces (e.g. grass and gravel). Twenty-six studies (81%) included physical measures with 25 (78%) reporting at least one distance, two (6%) reporting slope, four (12%) reporting the coefficient of friction and two (6%) reporting roughness. Only five of these studies (16%) discussed the measurement technique used, with four assessing the coefficient of friction with slip-meters or force plates and one measuring slope with a clinometer. Eleven studies (34%) gave only verbal descriptions of at least one of the surfaces tested.

### Non-physical surface measures (ratings, rankings and multiple-choice questions)

Only 80 of the 384 studies (21%) included non-physical surface measurements. These studies generally assessed more surfaces (mean of 7 where the number was specified) than studies that only used physical surface measures (3.5 surfaces). Non-physical surface measurements were highly varied across studies with no common questions or consistently used measures. The Supplementary material provides detail about the specific non-physical measures used in particular studies. Multiple measures were sometimes taken and measures included Likert rating scales (*n* = 31), continuous rating scales (*n* = 10), rankings (*n* = 6), multiple-choice questions (*n* = 9) and 2 choice questions (*n* = 5). Other methods used included self-reported descriptions or reports (*n* = 8) and focus group comments (*n* = 5). Forty-five of these studies (56%) assessed physical as well as non-physical surface measures.

### No surface measures (verbal descriptions only)

Twenty-nine of the 384 studies (8%) just described the surfaces verbally, with neither physical nor non-physical measurements provided. As an example, Wang et al. [18] reported data from five surfaces which were only reported as “walking flat, walking slope-up, walking slope-down, walking stairs-up and walking stairs-down” (page 4900). Some of these studies did specify the material of the surface (e.g. wood, snow, sand).

## Discussion

In this systematic review, we found that most studies provide inadequate information about walking surfaces. This makes it difficult to meaningfully compare findings between studies. While most studies (83%) in this review included physical measures of the surfaces that they tested, there was considerable variation in what was measured and how, whilst 8% of studies only described surfaces verbally, with neither physical nor non-physical measurements provided. In the introduction, we provided definitions of six surface types (stairs, slopes, slippery, compliant, rough and default). On the basis of the results of the systematic review, we now recommend how each of these surface types should be described in future studies.

- For every walkway (i.e. the route walked on a given surface), there should be:
  - ◦ At least one photograph of the surface (or the treadmill). Photographs should include an object of known size (such as a metre ruler) to indicate scale
  - ◦ A verbal description of the surface material
  - ◦ Dimensions of the physical extent of the surface (typically including length, i.e. the distance walked by the participant, plus width of the walkway)
  - ◦ The slope angle in degrees from the start to the end of the walkway (i.e. the angle from the horizontal: positive for up slopes, zero for horizontal surfaces and negative for down slopes)
  - ◦ (For stairs only) the number and width of the stairs, the height of the rise, the depth of the going and whether the stairs were traversed up, down or in both directions. More information will be required to describe non-standard stairs (for example, around corners or with landings)

Although few studies to date have included all this information it is easy to provide and so should be considered as a minimum requirement for reporting—even for studies which do not have a theoretical focus on comparing walking across different surfaces. Ideally, slipperiness, compliance and roughness should also be reported for every walkway. Unfortunately, there is, as yet, no consensus as to the best method for measuring these factors making it difficult to compare results across studies. For example, Powers et al. [15] found considerable variation in measurements of surface slipperiness (from coefficient of friction) between different tribometers [14].

Although few studies that we reviewed provided detailed physical surface measures, techniques used in other research areas could be employed. For example, studies from environmental science and palaeoanthropology have used data from laser imaging, detection and ranging (LIDAR) and geographic information system (GIS) to determine roughness in forests, on ice sheets and for bones (e.g. [3, 7, 9]. Such techniques could be adopted to measure roughness of walking surfaces. Other techniques include using accelerometer data to discriminate surfaces [6] and time-of-flight laser sensors to identify walking hazards such as pavement unevenness at a finer resolution (~ 0.25 cm) than traditional LIDAR methods (~ 15 cm) [8].

A potentially powerful approach would be to produce 3D surface models, for instance using photogrammetry (e.g. [1, 5, 16, 17]). Digital probe techniques can then be applied to these 3D surface models. For example, Costa et al. [5] used a portable device that captured surface information as participants walked over long stretches of pavements (700m). They created surface models from this data then calculated roughness from the models. Such techniques could be used to set up a database of surface measures for common materials (such as concrete, tarmac, grass) together with associated 3D models. There are analogous repositories of 3D surface models in other fields e.g. morphosource.org for the teeth and bones.

Our systematic review was limited to studies that compared multiple surfaces. It would be useful to repeat this review for studies of locomotion that tested only a single surface to assess whether, as we expect, these studies have similar inadequacies in the reporting of surfaces. Similarly, we excluded from our review studies that only tested stairs because this research area is distinct, well-developed and relatively self-contained. Given this, the reporting of surfaces may be superior for stairs-only studies compared to the generally low standard that we observed for the studies included in our review.

## Conclusions

A systematic review of 384 studies that investigated the influence of surface type on human walking and falling found that the majority of studies provided insufficient data about the physical and non-physical nature of the surfaces that they used. Surface descriptions were poor for all five categories of surface type that we considered (indoor, outdoor, treadmill, virtual reality, qualitative and default) and few studies specified what techniques were used to take measurements. We recommend that future studies provide, as a minimum, a verbal description of each surface used, together with a photograph and measurements of the surface length, width and slope. We suggest that, in addition, routinely providing 3D surface models together with standard measures of roughness, slipperiness and compliance, would aid making meaningful comparisons across studies. To assist in achieving this aim, we highlight developments in surface measurement in other fields that could be employed to improve the quality and consistency of surface descriptions in research on human locomotion.

### Supplementary Information

**Additional file 1: Table SM1.** PRISMA Checklist. Note that PRISMA primarily focusses on reviews that evaluate the effects of interventions. This was not our focus and therefore some sections of the PRISMA checklist did not fit our review. Specifically, we did not analyse the results sections of any of the studies in our review (we were only concerned with the method sections) and so we have put not applicable (n/a) for some items. **Table SM2.** Search terms used to search the electronic databases for the systematic review. *denotes a wildcard that was included in the search terms in order to include a variety of suffixes (e.g. child/children, rough/roughness). **Table S3.** Cohen’s kappa score for reviewer agreement for article inclusion and exclusion. Reviewer agreement. We calculated Cohen's kappa to measure reviewer agreement for inclusion and exclusion of articles in the initial screen, based on a random selection of over 10% of the articles. **Table S4.** Full Screen Table

## Data Availability

The supplementary materials for this paper include a spreadsheet detailing the information on the studies that is reported in this paper.
